# Progress in Surgery

**Published:** 1899-08-05

**Authors:** 


### Progress in Surgery.

### TUMOURS.

(*Continued from page* 298.)

**Treatment of Mammary Cancer.**—Watson Cheyne[1] has again written on this subject. He traces the further fate of the cases published three years ago in the Lettsomian Lectures, and adds other cases, in all of which the extensive (complete) operation was performed. Of the 37 patients who lived after the operation, 19 (or over 50 per cent.) have remained free from recurrence for over three years. As illustrating the value of the three years' limit as a test of success in operating, it is pointed out that only one of the 11 cases which three years ago had passed that limit has since shown signs of fresh disease, and it is doubtful whether that was a recurrence or a new development. Further, of 20 patients who had been alive and well over a year (one to six years) after the operation, 17 still remain well. This shows the great chance of freedom which patients alive and well a year after the operation possess after the thorough procedure advocated. Some points in regard to the operation are discussed. Owing to the danger of auto-infection if any incision is made into the cancerous tissue, the rule is laid down that when a swelling is of doubtful nature it should be excised with an area of apparently healthy tissue around, and should only be cut into after its removal from the body and the vicinity of the operation. Cheyne has seen, more than once, diffuse cancerous infection of a wound, more especially where cancerous glands have burst during removal. A small sponge should be stuffed into the wound made as above, which is then closely sutured, and the parts around and the hands of the operator disinfected. Stress is laid upon wide skin incisions, and these should not go straight down to the muscles. The skin, with just sufficient fat to maintain vitality, should be dissected up to the clavicle, middle line, and to the latissimus dorsi. The fascia over the pectoralis minor must be removed, as well as that over the pectoralis major. In removing glands the question of opening up the posterior triangle arises. Halsted always does so. Cheyne does not unless he finds that there are infected glands in the fat which runs upwards behind the axillary vessels. Halsted found evidences of infection in the fat and glands of the posterior triangle in 34 per cent. of a number of cases in which this tissue was particularly investigated. Cheyne does not regard the existence of enlarged glands in the posterior part of the posterior triangle as contra-indicating operation, but would not operate if there were glands of considerable size in front of or beneath the sterno-mastoid. In the after-treatment it is better to fix the arm away from the side, almost or quite at a right angle, by means of an axillary pad.

Butlin has recently written on this subject.[2] He was led to adopt the extensive mode of operating through the writings of Halsted, whose methods he adopts, and of Heidenhain and Stiles. He alludes to the importance of Stiles' observation that the glands in the apex of the axilla, above the pectoralis minor, and under the clavicle may be diseased, while the pectoral glands are free. Following Halsted, the pectoralis major muscle is removed by Butlin, and the pectoralis minor divided near the coracoid; and he says this removal does not impair the movement of the arm, which is more affected by the area of skin taken away. Of 13 cases operated upon more than three years ago 11 are alive and well, which is nearly 70 per cent. Butlin regards these results as exceptionally fortunate, and not likely to be equalled in another series of 13.

**The Dissemination of Cancer of the Breast.**—The operations just described were largely the outcome of the researches of Heidenhain and Stiles on this subject. Stiles[3] has recently again described his investigations. He points out that when cancers come under observation in all probability dissemination has taken place to a certain degree, and therefore as much of the tissue adjacent to the mamma which is liable to be involved in the dissemination should be removed. This is equivalent to the removal of the whole mamma and the tissues containing the lymphatics along which cancer cells may have passed, for Stiles' researches show that recurrence is much more likely to be due to cancer cells lurking in lymphatic spaces and left behind at the operation than to development of cancer again from remnants of mammary tissue in a precancerous or proliferative state. The importance of recognising the adeno-carcinomata (of Halsted) is urged. These tumours, though they have an encephaloid appearance and a tendency to fungate, are less malignant than ordinary cancer, and rarely give rise to secondary growths in the glands or to local recurrence, behaving more like duct-cancers. Stiles is inclined to think also that cancers developing in connection with mastitis are less malignant than ordinary cancers. Disseminated cancer cells have been found by Stiles both in the lymphatics of the mammary parenchyma—the perilobular, interlobular, periductal, and interacinous—also in the perivascular lymphatics. These vessels are generally larger than the small veins and arteries which they accompany. Again, Goldmann has shown that the small veins and arteries themselves are more prone to be affected than was thought. In Paget's disease, the disease begins at or near the mouths of the ducts, and spreads from thence both down the ducts and circumferentially in the skin, but why it spreads in foci limited to the epidermis is an unsolved question.

In recurrent nodules of cancer, however, usually no breast tissues can be found, the recurrence being in the extrinsic mammary tissues—the skin, circum-mammary fat, pectoral fascia, and muscle. The lymphatics of the mamma communicate with those of the skin by means of the suspensory ligaments of Cooper, and also through the rich sub-areolar plexus of Sappey, with which the cutaneous lymphatics freely communicate. Central tumours are specially liable to affect this areolar plexus, producing induration of the nipple and areola. In cancer *en cuirasse* the primary tumour is generally sub-areolar. But processes of parenchyma, terminating in fibrous prolongations, radiate from the circumference of the gland and pass insensibly into the connective tissue septa which separate the lobules of the circum-mammary fat, and cancerous foci are frequently found in these septa. Again, processes of parenchyma, analogous to those described above, pass from the deep surface of the mamma into the retro-mammary tissue—the pectoral fascia and muscle—which structures must therefore be removed if all breast parenchyma is to be taken away. Stiles asserts that in the majority of cases adhesion to the retro-mammary tissue takes place earlier than to the skin. Rotter is quoted as having found in rather more than a third of a series of carcinomatous breasts small cancerous nodules, visible to the naked eye, upon the posterior surface and in the substance of the pectoralis major, adjacent to the superior thoracic artery. Invasion of this retro-pectoral lymphatic tissue was found almost as frequently when the mamma was not adherent to the muscle as when it was so adherent. It is therefore recommended to remove both pectorals in all cases. Stiles also makes interesting remarks upon the dissemination of cancer in the axillary glands. Many glands are found to resemble fat lobules because fatty growth has invaded the hilum, leaving only a crescent of lymphoid tissue beneath the capsule. There is evidence that such glands may be developed from fat lobules; no doubt in others the change is one of functional involution. They are as liable to be infected with cancer as ordinary glands. Cancerous afferent and efferent lymphatics are not as frequently met with in the axilla as would be expected. In the so-called "thickening" of the lymphatics in the axilla, which surgeons often describe, Stiles asserts that the cords are in reality not lymphatics but ducts of the breast tissue running upon the surface of the serratus—the ducts of the axillary tail of the mamma. It is recommended to remove some of the fibres of the serratus in order to remove its fascia the more completely. Also, the sheath of the axillary vein should be removed, and the branches of the vein which proceed towards it from the chest. The subscapular nerves are to be spared, if possible.

[1] Lancet, Mar., 1899. [2] Brit. Med. Jour., Dec., 1898. [3] Brit. Med. Jour., June, 1899.

